# Ambushing the ambush hypothesis: predicting and evaluating off-frame codon frequencies in Prokaryotic Genomes

**DOI:** 10.1186/1471-2164-14-418

**Published:** 2013-06-22

**Authors:** David W Morgens, Charlotte H Chang, Andre RO Cavalcanti

**Affiliations:** 1 Biology Department, Pomona College, 175 W 6th st, Claremont, CA 91711, USA; 2 Department of Ecology and Evolutionary Biology, Princeton University, Princeton, NJ, USA

**Keywords:** Ambush hypothesis, Hidden stop codons, Codon bias, Markov models, Translational frameshifts

## Abstract

**Background:**

In this paper, we address the evidence for the Ambush Hypothesis. Proposed by Seligmann and Pollock, this hypothesis posits that there exists a selection for off-frame stop codons (OSCs) to counteract the possible deleterious effects of translational frameshifts, including the waste of resources and potential cytotoxicity. Two main types of study have been used to support the hypothesis. Some studies analyzed codon usage and showed that codons with more potential to create OSCs seem to be favored over codons with lower potential; they used this finding to support the Ambush Hypothesis. Another study used 342 bacterial genomes to evaluate the hypothesis directly, finding significant excesses of OSCs in these genomes.

**Results:**

We repeated both analyses with newer datasets and searched for other factors that could explain the observed trends. In the first case, the relative frequency of codons with the potential to create OSCs is directly correlated with the GC content of organisms, as stop codons are GC-poor. When evaluating the frequency of OSCs directly in 1,976 bacterial genomes we also detected a significant excess. However, when comparing the excess of OSCs with similarly obtained results for the frequency of out-of-frame sense codons, some sense codons have a more significant excess than stop codons.

**Conclusions:**

Two avenues of study have been used to support the Ambush Hypothesis. Using the same methods as these previous studies, we demonstrate that the evidence in support of the Ambush Hypothesis does not hold up against more rigorous testing.

## Background

**Figure 1:**
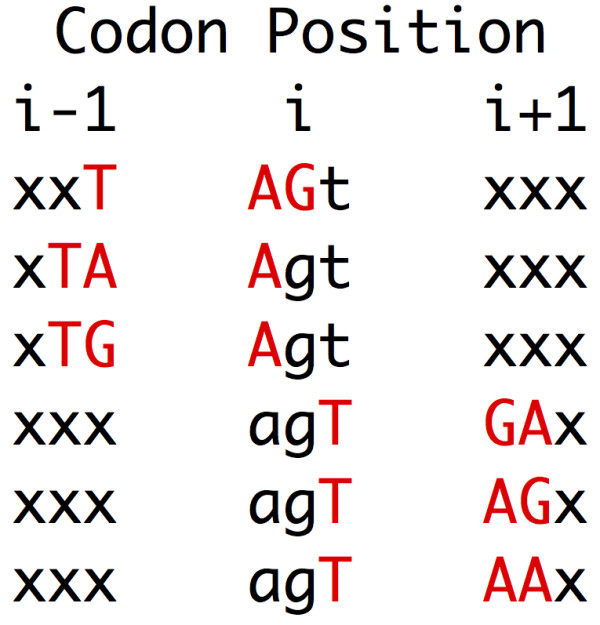
**Example of OSC formation.** There are six ways in which an arginine AGT codon at position i can form OSCs with codons at position i-1 or i+1. OSCs are given in upper case and red. X represents any base for the codons in positions i-1 and i+1.

Off-frame stop codons (OSCs), referred to variously as hidden stop codons, ambush codons, or premature stop codons (PSCs), are stop codons in the +1 and +2 reading frames of coding genes [1-4]. An OSC is formed from two consecutive in-frame sense codons, and different sense codons are able to form OSCs in zero to six different ways (Figure 1).

If a frameshift occurs during translation, the ribosome will continue to translate the mRNA in the wrong reading frame and to extend the protein by incorporating incorrect amino acids until a stop codon is reached in the new reading frame. Such events are, at best, a waste of the cell’s resources and, at worst, could result in a cytotoxic product. Given the potential deleterious effect of translational frameshifts, Seligmann and Pollock [1] proposed a mechanism by which these effects can be minimized. The Ambush Hypothesis [1] posits that having numerous OSCs would both minimize the waste of resources and reduce the chances of any cytotoxic products from a translational frameshift, and that as such, genomes with a large number of OSCs would be selectively favored. OSCs has also been reported to affect developmental stability in vertebrates [5] and protein expression levels [6].

It has been suggested that a similar selection to minimize the effects of translational frameshifts might have affected genetic code evolution and that the amino acid assignments in the code were optimized to generate a high number of OSCs [7]. The Ambush Hypothesis, however, predicts a continued selective force in favor of OSCs [1]. Thus, if this hypothesis were correct we would expect to find more OSCs than expected by chance in coding regions of genomes.

Support for the Ambush Hypothesis has relied on two methods to evaluate the excesses of OSCs in contemporary genomes. The first methodology relies on the fact that some codons are more likely to generate OSCs than others, and that these codons should be favored, leading to a higher number of OSCs. This prediction was originally tested in 100 organisms [1] and a similar analysis expanded the dataset to over 14,000 organisms [3].

These studies showed mixed results. In particular, the most recent analysis [3] found that only six percent of organisms – 901 out of 14,468 – showed a significant positive correlation between the usage of a codon and its potential to form OSCs. However, in both studies the authors concluded that the existence of any significant positive correlations indicated selection for OSCs [1,3].

We believe that the data used in these studies is not appropriate to test the Ambush Hypothesis. Codon usage bias is known to vary between organisms and several hypotheses have been proposed to explain these differences [8]. The selection for OSCs predicted by the Ambush Hypothesis should be a very weak selective force as translational frameshifts affect a single protein product at a time, and regardless of the hypothesis, some level of OSCs is expected by chance. The number and strength of other selective pressures influencing codon bias makes it unlikely that codon usage can be used to evaluate the Ambush Hypothesis. In fact, as we will show, the usage of codons with high potential to form OSCs can be almost completely explained by the GC content of genomes.

The second methodology used to test the Ambush Hypothesis is more direct and compares the actual frequency of OSCs in a genome with the frequency expected by chance based on the coding properties of that genome [4]. In a 2010 study [4], the authors used second and fifth-order three-periodic Markov models to calculate the expected number of hidden stops in 342 phylogenetically representative prokaryotes. Comparing these to the actual counts of OSCs in these genomes, they found a statistically significant excess of OSCs in 93% of the organisms examined. In addition, the authors tested another prediction of the Ambush Hypothesis.

Specifically, as stop codons are AT-rich, GC-rich genomes should have fewer OSCs simply by chance. If the Ambush Hypothesis were correct, the pressure to have OSCs would be stronger in GC-rich organisms; in comparison, AT-rich organisms would inherently have a higher occurrence of OSCs and therefore experience reduced selective pressure for having extra OSCs. Thus, the proportional excesses of OSCs should be larger in GC-rich organisms than in AT-rich ones. The authors tested this prediction and found a correlation between the observed excess of OSCs and GC content, as expected if the hypothesis were correct. They interpreted their results as strong evidence in favor of the Ambush Hypothesis [4].

However, one has to be careful when interpreting statistical significance in biological systems. While 93% is an impressive and statistically significant majority, this number is given without context. With no controls or theoretical framework to compare the 93% to, it is difficult to judge the biological significance of the results.

In this paper, we used 1,976 complete bacterial transcriptomes to reassess the evidence for the Ambush Hypothesis using both previously described methodologies. We first establish that codon usage is an irrelevant measure of the effects of the Ambush Hypothesis, as the usage of codons with high potential to form OSCs can be almost completely explained by the GC content of genomes. We then evaluate the excess of OSCs in the transcriptomes directly by comparing the observed frequency of OSCs with the expected frequency determined using Markov models. Although our results show the same highly significant statistical excess of OSCs in the organisms studied, they also show that several sense codons are present out-of-frame with similar or even greater excesses than the stop codons are. These results show that neither of the previously used approaches can be used as evidence in favor of the Ambush Hypothesis.

## Results and discussion

### Codon usage analysis

A previous study correlated codon usage with the potential to form OSCs for 14,468 organisms [3]. The study used codon usage data from the CUTG (Codon Usage Tabulated from GenBank) database [9]. This database contains codon usage information for all full-length protein gene entries present in GenBank tabulated by organism, regardless of the number of sequences for the organism. For some organisms, those with fully sequenced genomes, the database contains an accurate estimate of codon bias; for other, those with just a few sampled genes, the real codon usage might be very different from that reported in the database. This is by design; the CUTG database tries to be comprehensive and to summarize all the data available in GenBank [9]. In the current study we chose to be conservative and included only those prokaryotic organisms that have a full transcriptome sequence available on GenBank, which limits us to 1,976 organisms.

The previous study found that 901 of 14,468 (6.2%) organisms possessed significant (p <0.05) positive correlations between codon bias and potential to form OSCs [3]. Neither this study [3] nor a previous analysis of 100 organisms [1] present convincing evidence of their conclusive statements: that there exists a positive correlation between potential to create OSCs and codon bias.

When we tested the validity of this statement in the dataset of fully sequenced bacterial genomes, we found statistically significant (p < 0.05) positive correlations between codon usage and the propensity of that codon to form OSCs in 668 of the 1,976 genomes analyzed (34%). Assuming, as previous papers have, that a selection for OSCs influences codon usage, this could be interpreted as evidence for the Ambush Hypothesis [1,3].

**Figure 2:**
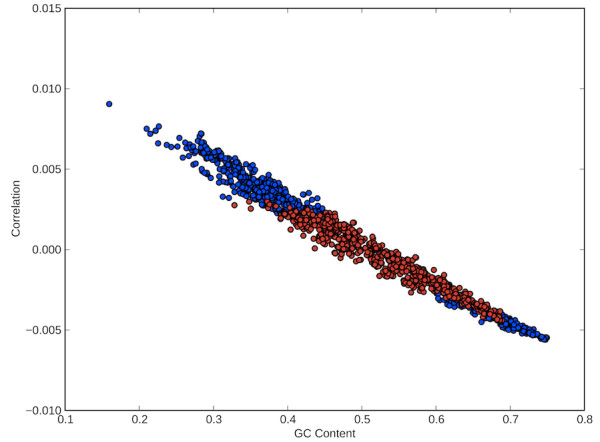
**GC content versus slope of regression between potential to form OSCs and usage of a codon.** A linear regression gives the line y = -0.0251x + 0.013 with R^2^ = 0.978, indicating that GC content explains 97.8% of the variation in the correlations. Organisms in which the correlation between a codon usage and its potential to form OSCs are positively or negatively significant (p < 0.05) are given in blue, while those with non-significant correlations are given in red. The Ambush Hypothesis predicts that organisms should have significant positive correlations between the usage of a given codon and its potential to form OSCs. There are no significant positive correlations in organisms with GC content higher than 46% and only three non-significant correlations in organisms with GC content lower than 37%.

To determine if this assumption is justified, we searched for other potential explanations of the observed correlation, specifically the impact of GC content on codon usage. When we compared the linear slope of each of these correlations to the GC content of the organisms, we found a strong linear relationship (R^2^ ~ 0.978) where a higher GC content was associated with a more strongly inverse relationship between codon usage and its ability to form OSCs (Figure 2). This implies, among other things, that the connection between codon bias and the potential for OSCs is no more than a reflection of GC content alone. In fact, there are no significant positive correlations in organisms with a GC content higher than 46% and only three insignificant correlations in organisms with GC levels below 37% (Figure 2).

The correlation between codon usage, potential to form OSCs, and GC content can be explained by the fact that stop codons are AT-rich. Thus, codons that have the potential to form OSCs tend to be AT-rich and consequently are disproportionately prevalent in organisms with AT-rich genomes. Given that other selective forces or mutational biases determine the GC content of an organism [8,10-12], this implies that the relationship found between a codon’s usage and its propensity to form OSCs is a poor test of the predictions of the Ambush Hypothesis, as it is easily explained by the high AT content of stop codons.

### Excesses of OSCs in fully sequenced bacterial genomes

Even though it seems like selection for OSCs doesn’t affect codon usage, it is possible that it could affect the frequency with which one codon follows another in the genome. This could lead to an excess of OSCs over the expected value for a genome with the same coding properties. The authors in [4] tested this prediction of the model by analyzing the genomes of 342 prokaryotes.

For each organism, they counted the number of OSCs in all transcripts and compared it to the frequency of OSCs expected from randomized genomes, which were constructed to maintain the same coding properties of the original genome. To generate the randomized genomes they used both second-order and fifth-order, 3-periodic Markov models implemented in the MARKOV package of GenRGenS [4,13]. These models generate random genomes that preserve the dinucleotide or pentanucleotide frequencies of the original genome respectively.

**Figure 3:**
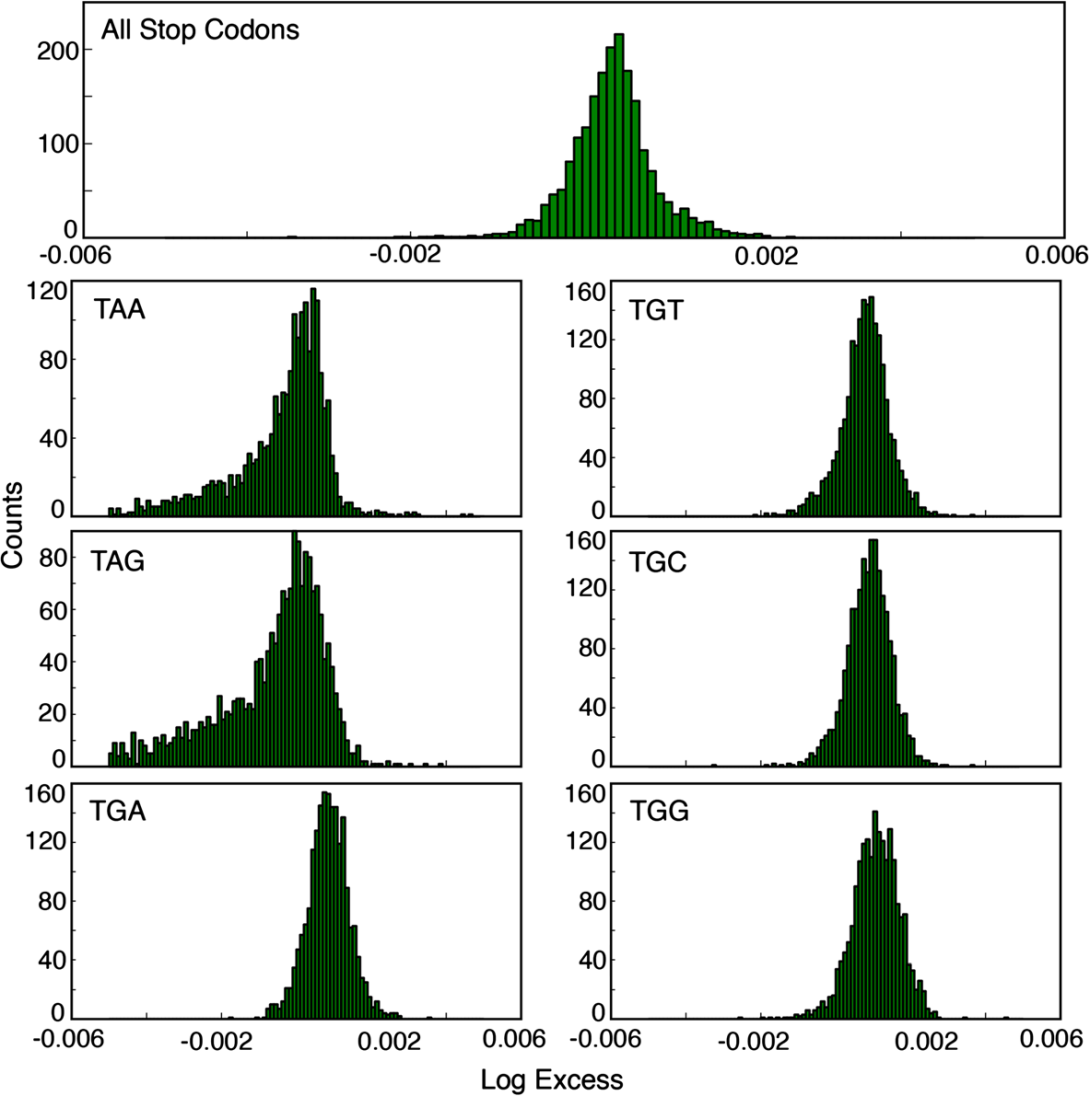
**Histograms of percent excess of selected out-of-frame codons in the 1,976 bacterial genomes analyzed for the 2–3 Markov model.** The first graph shows the percent excess in the observed number of all stop codons out-of-frame. The other graphs show the percent excess for the 3 stop codons TAA, TAG and TGA; and for 3 TGN codons, TGC, TGG, and TGT. For the stop codons, only out-of-frame TGA codons have a systematic excess in the organism studied. All out-of-frame TGN codons have a systematic excess and are present out-of-frame in larger excesses than the set of all stop codons. The 5–3 Markov model gives similar results (data not shown).

Tse et al. [4] report a significant excess of OSCs when compared to the expected frequency of OSCs in 93% of the genomes analyzed. In our results, we found that 83% of the 1,976 genomes analyzed have a significant excess of OSCs for both the second-order and the fifth-order Markov models. They also report that in no genome did the observed frequency of OSCs exceed the expected value by more than 6% [4]. In our own analysis, no organism was found with an excess greater than 1% (Figure 3).

To consider the magnitude of the observed effect, Tse et al. [4] calculated that, based on their results, *Yersinia entercocolitica* has about 800 OSCs more than expected by chance [4]. This number sounds high until one considers that *Y. enterococolitica* has over 4,000 coding sequences and that the excess comes to about 1/5 of an OSC per gene. *Y. enterococolitica* has 168,841 OSCs in its transcriptome, 112 more than expected under our 2–3 Markov model, which amounts to an extra 0.028 OSCs per gene. Even though these numbers are low, both results are highly statistically significant. Considering that the expected selection for OSCs should be an extremely weak force, these results seem to validate the Ambush Hypothesis.

Yet without a theoretical framework how can we determine whether a statistically significant excess is biologically meaningful? Nature provides such a framework in the form of 61 sense codons. They provide a context that can be used to evaluate the biological significance of the observed excesses of OSCs, assuming that there is no selection for off-frame sense codons.

In light of this context, OSCs are indistinguishable from other off-frame codons. While the excesses of OSCs do tend to be among the highest observed, TGN sense codons show excesses that are even more statistically significant than those of stop codons (Table 1). When one considers the excesses of individual stop codons, only TGA shows a systematic excess (Figure 3), yet all TGN codons show excesses comparable to those of TGA (Figure 3).

**Table 1:** Summary of the results of the Markov models for selected codons

**Algorithm**	**2-3 Markov model**				**5-3 Markov model**			
**Codons**	**% organisms with excess**^**a**^	**Average log ratio**	**Correlation value**	**R value**	**% organisms with excess**^**a**^	**Average log ratio**	**Correlation value**	**R value**
TGG	96	0.0011	0.003	0.58	96	0.0011	0.003	0.51
TGC	93	0.0009	0.002	0.45	93	0.0009	0.002	0.45
TGT	90	0.0008	0.002	0.31	90	0.0008	0.002	0.29
TGA	94	0.0008	0.002	0.46	93	0.0008	0.002	0.40
TAG	38	-0.0009	-0.009	-0.58	39	-0.0011	-0.012	-0.62
TAA	47	-0.0005	-0.007	-0.50	44	-0.0008	-0.010	-0.61
All Stop Codons	85	0.0005	0.001	0.31	83	0.0004	0.001	0.20

^a^ Percent of the 1,976 organisms tested with excess of the codon off-frame.

Thus, interpreting the statistically significant excesses of OSCs as evidence of selection in favor of OSCs would impel us to interpret our data as evidence for the selection of other off-frame sense codons as well. For example, under this framework, our data provide compelling evidence of selection pressure for off-frame TGN codons, and that this selective force is stronger than that operating on OSCs (Table 1).

Perhaps the most convincing piece of evidence previously brought forth to support the Ambush Hypothesis is the positive relationship between GC content of a genome and its excess of OSCs [4]. This is a validation of a specific prediction of the Ambush Hypothesis: In organisms with high GC content, fewer OSCs are expected by chance, thus a greater excess is needed to counterbalance the effects of translational frameshifts.

We can replicate this result with our own data, but again we must interpret this in context. Do other codons show similar relationships to GC content? In fact, they do: using both Markov models, 10 individual sense codons show a stronger (steeper slope and larger R^2^-values) positive relationship between GC content and off-frame occurrence than OSCs.

**Figure 4:**
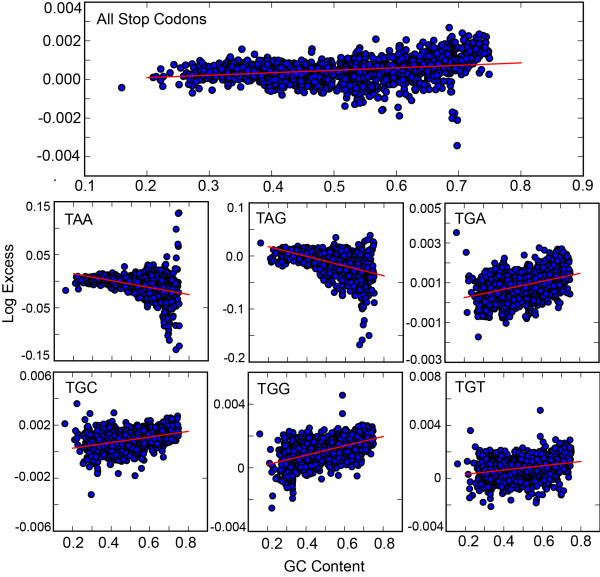
**Correlation between GC content of genomes and excess of certain off-frame codons calculated using the 2–3 Markov model.** The top graph shows the correlation between the GC content and the excess of OSCs in the organisms studied; as predicted by the Ambush Hypothesis this graph has a significantly positive correlation. The graphs in the second row show the correlation between the GC content and the excess with which each stop appears out-of-frame; it should be noted that only TGA shows a positive correlation as predicted by the hypothesis, while TAA and TAG show negative correlations in opposition to the hypothesis’ prediction. The graphs in the third row show the same correlation but for the TGG, TGC and TGT codons; all three of these codons show significant positive correlations, with TGG and TGC showing even stronger and more significant correlations than TGA. The 5–3 Markov model gives similar results (data not shown).

Furthermore, TGA is the only stop codon to clearly demonstrate a positively linear relationship with GC content; the other stop codons, TAA and TAG, actually present an inverse relationship between GC content and the excess of the codon in out-of-frame positions. Interestingly, the TGN codons show positive correlations with GC content, and the correlation coefficients for TGG and TGC are even larger and more significant than that for TGA (Figure 4).

Taken together, our results suggest that the previous results in support of the Ambush Hypothesis were simply detecting excesses of TGA codons. While this could still be interpreted as evidence for the Hypothesis, it seems to stem from an unrelated statistically significant overrepresentation of TGN codons off-frame. We will not speculate here if this statistically significant excess of out-of-frame TGN codons is biologically relevant, but it is not consistent with the Ambush Hypothesis.

## Conclusion

Previous papers employed the correlation between a codon’s usage and its propensity to form OSCs as evidence for the Ambush Hypothesis [1,3]. We have shown that this data reflects only the GC content of the organism and thus cannot be used to evaluate the hypothesis.

Tse et al. found an excess of OSCs in 93% of 342 bacterial genomes using Markov models and also detected a significant positive correlation between the GC content of a genome and the OSC excess of the genome, consistent with the predictions of the Ambush Hypothesis [4]. In an expanded dataset comprising 1,976 bacterial genomes, we observed an excess of OSCs in 83% of the organisms and demonstrated a strongly positive relationship between genome GC content and OSC excess. However, we also found that an excesses of three sense codons are present in more organisms and at a higher frequency than stop codons, and are are even more strongly correlated with GC content (Table 1). Given that the Ambush Hypothesis does not predict any selection for off-frame sense codons, we conclude that our results and the analogous results by [4] do not represent evidence in favor of the Hypothesis.

## Methods

### Genomes

We retrieved the annotated transcripts of 2,023 bacterial genomes available from NCBI on August 2012 (http://ftp.ncbi.nlm.nih.gov/genomes/). We excluded from the analyses organisms that reassign stop codons, and for the remaining 1,976 organisms we removed incomplete or ambiguous transcripts.

### Codon usage analysis

Previous analyses [1,3] tried to verify the Ambush Hypothesis by correlating the usage of each codon with the number of ways in which the codon can form an OSC – from 0 to 6 ways (Figure 1). Both analyses used the CUTG (Codon Usage Tabulated from GenBank) database to determine the usage of each codon in different organisms and did a linear regression to determine the correlation coefficient between codon usage and the number of ways in which it can form an OSC.

We replicate these analyses [1,3] using 1,976 fully sequenced bacterial genomes. Instead of using the CUTG database, we calculate the codon usage for each organism by directly analyzing the transcripts in the GenBank annotation. GC content for each organism was also calculated from the transcripts. We grouped each codon based on the number of ways it can form an OSC. Note that we do not include stop codons themselves in this analysis as none occur in-frame in the coding region. Subsequently, using the average codon usage of each genome, we calculated the correlation (Spearman’s rank coefficient) between codon bias and potential to form OSCs.

### Excesses of OSCs in fully sequenced bacterial genomes

We counted the number of OSCs in the +1 and +2 frames for each organism directly from available transcripts. These corresponded to our observed values, the true number of OSCs in each organism. To produce expected values of OSCs, we used the same two Markov models described in [4] to create artificial sequences that preserved the fundamental structure of the genome. These models have been used before to successfully identify microbial genes [14,15].

For each organism, we used the complete transcriptome to train our Markov model. The terminating stop codon was removed before training so that stop codons would not be inserted into our artificial genome in frame, as this does not occur biologically and would bias the calculation of the expected frequency of OSCs. The transition matrix generated this way contained the probability of each nucleotide following a given dinucleotide or pentanucleotide, which were used to parameterize the second and fifth order models.

To generate the artificial sequences, we initiate each artificial transcript using the first two or five nucleotides of the natural transcript and then use the probabilities dictated in the transition matrix to add nucleotides one by one, ending when the artificial transcript was an equivalent length to the natural transcript. We then counted the number of OSCs in the entire artificial genome. We repeated this 200 times for each organism to calculate an average expected value. Increasing the number of repetitions did not affect the average significantly. These expected counts were compared to the actual values.

We repeated this procedure using one codon at a time to calculate the overrepresentation with which each codon appeared off-frame in the transcripts of the genomes analyzed. For each organism, after calculating both the expected and the observed off-frame frequencies for the three stop codons and for all 64 codons individually, we quantified the over- or under-representation of a codon (or stop codons) using the natural log of the ratio of the number of observed off-frame codons over the average number of predicted off-frame codons. If this log-ratio was negative, then there were less off-frame codons than expected. If it was positive, then there were more off-frame codons than expected. A log ratio was used because it removes the inherent bias of ratios towards higher values.

There were minor differences between our basic analysis and that of [4]: Before training their Markov model, Tse et al. removed any transcript less than 100 amino acids long. We found this cutoff arbitrary and included all transcripts. They also limited their analysis to one species per genus to prevent oversampling [4]. Given the large number of organisms available to us, we did not trim our dataset, reasoning that the dangers of oversampling were less important and less likely to occur than the inadvertent introduction of bias. All analyses were performed using Python scripts. Source code is available upon request.

## Competing interests

The authors declare that they have no competing interests.

## Authors’ contributions

DWM, CHC and AROC participated in all steps of the analyses. All authors read and approved the manuscript.
